# Sin Nombre Virus as Unlikely Reverse Zoonotic Threat

**DOI:** 10.3201/eid3102.241532

**Published:** 2025-02

**Authors:** Jérémie Prévost, Nikesh Tailor, Anders Leung, Bryce Warner, David Safronetz

**Affiliations:** Public Health Agency of Canada, Winnipeg, Manitoba, Canada (J. Prévost, N. Tailor, A. Leung, B. Warner, D. Safronetz); University of Manitoba, Winnipeg (D. Safronetz)

**Keywords:** Hantavirus, Sin Nombre virus, zoonotic disease, rodentborne pathogens, zoonoses, viruses, Canada

## Abstract

We inoculated clinical materials into deer mice to attempt isolation of Sin Nombre virus. We did not observe productive infection in the natural rodent reservoir. Genomic comparisons between rodent reservoirs and human disease may provide insight into hantavirus evolution and genetic determinants, but reverse zoonosis of Sin Nombre virus appears unlikely.

Sin Nombre virus (SNV) is the primary cause of human hantavirus cardiopulmonary syndrome (HCPS) in North America. In nature, *Peromyscus maniculatus* deer mice are the reservoir host for SNV, although other rodents may also serve as competent reservoir hosts (*1*). Human HCPS is characterized by a sudden onset of respiratory distress that rapidly progresses and requires urgent medical attention.

As for many hantaviruses, SNV isolation has proven challenging. The extended prodromal phase, often >14 days, precludes collection of optimal samples with peak viral titers and minimal host immune responses for virus isolation. In previous studies, Andes virus (ANDV) was isolated from serum samples fortuitously collected immediately before HCPS disease onset, as well as from oral, nasal, or urine specimens (*2*,*3*). Those detections were likely achievable because of ANDV’s ability to transmit from human to human, an attribute not known in SNV, and higher viral burdens in mucosal specimens of patients infected with ANDV (*4*).

Samples submitted for diagnostic confirmation of HCPS are collected after symptom onset and commonly include only serum or whole blood. In Canada, hantavirus diagnostic testing is done through a combination of serologic and molecular testing at the National Microbiology Laboratory of the Public Health Agency of Canada (*5*). By 2024, >150 cases of HCPS in Canada had been confirmed. Despite efforts to propagate SNV from acute samples, isolation attempts on standard Vero cell culture have been unsuccessful. We previously showed that Vero cell propagation alters the virulence of SNV in nonhuman primates (NHPs) and infectivity in deer mice (*6*,*7*). We sought to assess whether direct inoculation of deer mice with clinical material would enable isolation of virus without prior Vero propagation.

We inoculated laboratory-reared deer mice (*Peromyscus maniculatus rufinus*, both sexes, >4 weeks of age, 3–6 per group) via intraperitoneal injection with acute serum from laboratory-confirmed symptomatic HCPS (n = 10) case-patients, SNV-infected NHPs with HCPS (n = 5), or cell culture supernatant containing Vero-propagated SNV (n = 2) (Table; Appendix). Acute specimens from HCPS case-patients were positive for SNV by reverse transcription PCR (RT-PCR), were IgM positive, and had low or no detectable IgG against hantaviruses in serum. NHP samples infected with the deer mice–only passaged SNV (*6*) were collected immediately before or shortly after apparent signs of disease. Those samples were positive by reverse transcription PCR (RT-PCR) and IgM­-positive by serology but also had detectable IgG. When possible, serum from HCPS case-patients was inoculated into deer mice without a freeze-thaw cycle. Serum from NHPs and the SNV cell culture supernatant were previously cryopreserved.

**Table:** Results of experimental inoculation using deer mice in a study of SNV as an unlikely reverse zoonotic threat*

Sample ID	Original infected sample							Experimental infection of deer mice					
	Origin	qRT-PCR, relative Ct value†			Serology, endpoint titer‡			qRT-PCR, positive/total samples†					Serology, endpoint IgG titer§
		Blood	BAL, others		IgM	IgG		Lung	Liver	Spleen	Kidney	Blood	
HAN67/23	Human	22.9	SNS		>6,400	–		0/3	NA	NA	NA	0/3	3/3 (100)
HAN79/23	Human	25.0	29.2		1,600	–		0/3	NA	NA	NA	0/3	2/3 (100–1,600)
HAN124/23	Human	31.5	28.4		>6,400	–		0/4	0/4	0/4	0/4	0/4	4/4 (400–1,600)
HAN126/23	Human	+	SNS		1,600	–		0/4	0/4	0/4	0/4	0/4	4/4 (100–400)
HAN238/23	Human	+	SNS		1,600	400		0/3	0/3	0/3	0/3	0/3	N/A
HAN018/21	Human	28.0	SNS		400	–		0/4	0/4	0/4	0/4	0/4	4/4 (100–400)
HAN176/22	Human	25.0	SNS		1,600	–		0/4	0/4	0/4	0/4	0/4	4/4 (400–1,600)
HAN173/23	Human	+	29.5		>6,400	–		0/4	0/4	0/4	0/4	0/4	4/4 (100–1,600)
HAN194/23	Human	27.7	SNS		1,600	400		0/4	0/4	0/4	0/4	0/4	4/4 (400–1,600)
HAN266/23	Human	28.1	SNS		>6,400	100		1/4	1/4	0/4	0/4	0/4	4/4 (100–400)
Total human samples								1/37	1/31	0/31	0/31	0/37	33/34
EC983	NHP	24.3	27.1		1,600	1,600		0/6	0/6	0/6	0/6	0/6	6/6 (100–1,600)
MB1599	NHP	23.4	27.2		400	800		0/4	0/4	0/4	0/4	0/4	4/4 (400)
EC1545	NHP	24.1	28.5		3,200	1,600		0/4	0/4	0/4	0/4	0/4	3/4 (100–400)
MB1291	NHP	25.6	>35		400	400		0/4	0/4	0/4	0/4	0/4	4/4 (100–400)
NV1021	NHP	24.0	30.5		800	800		0/4	0/4	0/4	0/4	0/4	4/4 (100–400)
Total NHP samples								0/22	0/22	0/22	0/22	0/22	21/22
Vero-adapted SNV 77734	VCL prep 1	NA	22.0		NA	NA		3/3	2/3	3/3	3/3	2/3	NA
	VCL prep 2	NA	17.5		NA	NA		4/4	3/4	4/4	4/4	0/4	4/4 (>6,400)
Total Vero samples								7/7	5/7	7/7	7/7	2/7	4/4

*Deer mice (*Peromyscus maniculatus*) were inoculated with specimens from SNV-infected humans, cynomolgus macaques (*Macaca fascicularis*), and Vero cells. Endpoint titers are measured in reciprocal serum dilution. BAL, bronchoalveolar lavage; Ct, cycle threshold; NA, not available; NHP, nonhuman primate; OD, optical density; qRT-PCR, qualitative reverse transcription PCR; SNS, sample not submitted; SNV, Sin Nombre virus; VCL, Vero cell line; +, RT-PCR–positive sample but Ct value not available; –, not detected. †qRT-PCR threshold: Ct <35. ‡Seropositivity threshold: >0.6 net OD_405_. §Seropositivity threshold: mean OD_650_ from negative control + 3 SD (Appendix).

At 14 days after infection, when SNV in experimentally inoculated deer mice is readily detectable in multiple organs (*7*,*8*), we euthanized the mice and collected samples (blood, serum, lung, liver, spleen, kidney) for analyses. Deer mice inoculated with serum from HCPS case-patients or experimentally infected NHPs tested negative for SNV RNA. All but 1 animal had detectable IgG against the nucleocapsid protein and showed exposure to SNV (Table). Another animal inoculated with human serum had low levels of SNV detected in lung and liver specimens, although strand-specific RT-PCR could not detect antigenome RNA (Appendix Table), suggesting inoculum-derived infection. All 7 deer mice injected with Vero-propagated SNV had multiple SNV-positive tissues, which is contradictory to our previous findings (*7*). Strand-specific quantitative RT-PCR confirmed the presence of replicating SNV; however, the presence of replicating SNV was 2–4 logs less than comparable tissues from mice inoculated with deer mice–only passaged SNV (Appendix Table).

The original aim of this study was to create a reliable method to isolate hantaviruses from clinical materials from confirmed HCPS cases by using natural reservoirs. However, after attempting that approach with samples from 10 unique HCPS case-patients and material from 5 NHPs experimentally infected with an SNV strain originally isolated from the deer mice colony founders, developing that method does not seem possible, at least not as outlined here (Figure). Although we did not determine SNV-neutralizing titers, the lack of IgG response indicates that all clinical samples were likely not completely neutralized before inoculation of deer mice. Nevertheless, this work addresses an overlooked aspect of hantaviruses: the potential ability to spillback and create reverse zoonotic events. Our work suggests that spillback is unlikely, at least for SNV, which implies that humans are truly dead-end hosts of SNV. Thus, virus evolution is primarily, if not exclusively, occurring in the natural rodent reservoirs.

**Figure:**
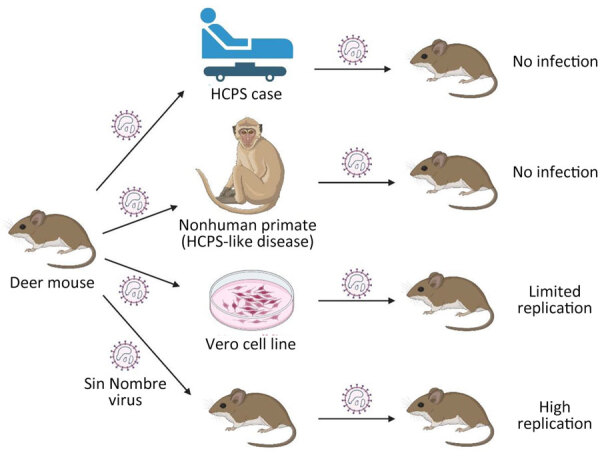
Experimental infection of North American deer mice (*Peromyscus maniculatus*) with Sin Nombre virus (SNV) to determine whether direct inoculation with clinical material would enable isolation of virus without prior Vero propagation. Infection of humans or nonhuman primates with deer mouse–derived SNV causes HCPS. This study shows that SNV retrieved from HCPS cases or infected nonhuman primates does not generate a productive infection in deer mice. SNV can also infect the Vero cell line upon passaging and adaption, but it reduces its infectivity in deer mice compared with deer mouse–only passaged SNV. The figure was prepared using images from BioRender.com (https://www.biorender.com). HCPS, hantavirus cardiopulmonary syndrome.

In conclusion, genetically, hantaviruses have proven difficult to adapt in disease modeling efforts, and only rodent-derived isolates or inocula have recapitulated human disease in hamsters and NHPs (*6*,*9*,*10*). The molecular determinants of virulence are largely unknown, and without a reverse genetics system will be difficult to elucidate. Thus, to clarify hantavirus evolution and genetic factors associated with human disease, SNV genomic surveillance is needed, especially to elucidate hantavirus evolution and genetic factors associated with human disease.

Appendix Supplemental methods regarding Sin Nombre virus as an unlikely reverse zoonotic threat
