# Supplementary material for: Sin Nombre Virus as Unlikely Reverse Zoonotic Threat
Source: Emerg Infect Dis. 2025 Feb;31(2):385–8. doi: 10.3201/eid3102.241532 (PMC11845128; doi:10.3201/eid3102.241532)
Supplement: Appendix — Supplemental methods regarding Sin Nombre virus as an unlikely reverse zoonotic threat [file 24-1532-Techapp-s1.pdf]

Article DOI: https://doi.org/10.3201/eid3102.241532

EID cannot ensure accessibility for supplementary materials supplied by authors. Readers who have difficulty accessing supplementary content should contact the authors for assistance.

Sin Nombre Virus as Unlikely Reverse Zoonotic Threat

Appendix

Methods

Animal Ethics Statement

All experiments described were done at the National Microbiology Laboratory (NML) of the Public Health Agency of Canada. Experiments were approved by the animal care committee at the Canadian Science Center for Human and Animal Health in accordance with the guidelines set by the Canadian Council on Animal Care. The deer mice were provided by a breeding colony 0housed at the University of Manitoba (*1*). All the deer mice from the colony were seronegative and Sin Nombre virus free. All the incoming animals were acclimated for at least 1 week before the experimental procedures began.

Animal Infections

All the animal work and infections were performed under BSL-4 conditions at the NML. The animals were given food and water ad libitum and monitored daily throughout the course of the experiments. The deer mice were infected intraperitoneally with 100 µL of SNV-infected material from different origins: infected human or nonhuman primate (NHP) sera and Vero-adapted SNV 77734 or SNV 77734 that was passaged only in vivo within deer mice. Two weeks after infection, all the mice were euthanized, and blood and tissue samples (lung, liver, kidney, spleen) were taken to determine the presence of SNV and seroconversion. Tissue samples were homogenized in 600 µL of DMEM (Thermo Fisher Scientific), clarified by centrifugation, and inactivated in AVL lysis buffer (Qiagen). All RNA extraction was performed by using the QIAamp viral RNA Mini Kit (Qiagen) according to the manufacturer’s instructions.

Molecular Detection Assays

Molecular detection of the SNV S segment in RNA samples from human HCPS cases, NHPs, deer mice, or the Vero cell line was tested in a 1-step qRT-PCR assay, as previously described (*2*), using the following primers: SNV-S140-F 5′-AAKTGGACCCCGATGAYGTTAA and SNV-S212-R 5′-TTGGTYTCCAATGCA GACACA. The following probe was used: SNV-S163-P 5′-6FAM-AAAAGCACATTACAGAGCAGACGGGCAG. We used the TaqPath 1-Step Multiplex Master Mix (Thermo Fisher Scientific) according to the manufacturer’s instructions. Alternatively, a conventional RT-PCR was performed with the SuperScript III One-Step RT-PCR System with Platinum Taq DNA Polymerase (Thermo Fisher Scientific), according to the manufacturer’s instructions, using the following primers: SNV-S142-F 5′-TTGGACCCCGATGATGTTAACAA and SNV-S302-R 5′-TCAGGTTCAATCCCTGTTGGATCAA. The 161-bp fragment generated by RT-PCR was visualized by using FlashGel DNA Cassettes (Lonza Bioscience).

For the detection of SNV active replication, we used a strand-specific 2-step qRT-PCR assay able to detect SNV antigenome as adapted from (*3*). RNA derived from infected deer mouse tissues was reverse transcribed by using the SuperScript IV First-Strand Synthesis System (Thermo Fisher Scientific), according to the manufacturer’s instructions, with the SNV-S445-R primer 5′-GGATAATCGGTAATGCAAAACT to determine the presence and relative amount of positive-strand SNV S segment RNA. Two microliters of cDNA was removed for subsequent qPCR. Detection of SNV positive-strand S segment cDNA was tested by qPCR with the QuantiTect Probe PCR kit (Qiagen), according to the manufacturer’s instructions, using the following primers: SNV-S179-F 5′-GCAGACGGGCAGCTGTG and SNV-S245-R 5′-AGATCAGCCAGTTCCCGCT. The probe used was SNV-S198-P, 5′-6FAM-TGCATTGGAGACCAAACTCGGAGAACTT.

Serologic Assays

Assays used to determine the seroconversion of human HCPS cases has been previously described (*2*). Briefly, detection of IgG antibodies was done by using an ELISA assay with Black Creek Canal orthohantavirus (BCCV)–infected Vero E6 lysate as a positive antigen and mock-infected Vero E6 lysate as the negative antigen. The IgM detection assay is a mu-capture ELISA

using BCCV-infected and mock-infected Vero E6 cell slurries and a mouse monoclonal antibody directed against aa 66–78 of SNV nucleocapsid (US Biologicals).

The detection of IgG and IgM antibodies in SNV-infected NHP or deer mouse samples was performed by using an anti-nucleocapsid indirect ELISA assay [adapted from (*4*)]. Briefly, 96-well high binding plates (Corning) were coated with recombinant SNV nucleocapsid (Genscript) at 100 ng per well. Serum samples were serially diluted in blocking buffer (PBS + 0.1% Tween20 + 5% skim milk) starting with a 1:100 dilution. The following HRP-conjugated secondary antibodies were used: anti-human IgG and anti-human IgM antibodies (KPL) for NHP serum, and anti–*Peromyscus leucopus* IgG antibody (KPL) for deer mouse serum. For NHP samples, HRP activity was quantified by using the ABTS substrate (KPL) before reading the OD values at 405 nm. Positive samples were those that had an OD greater than 0.6 compared with negative control wells. For deer mouse samples, HRP activity was quantified by using the TMB substrate (Thermo Fisher Scientific) before reading the OD values at 650 nm. Positive samples were those that had an OD greater than the mean OD plus 3 standard deviations seen in the negative control wells.

References

1. Warner BM, Stein DR, Griffin BD, Tierney K, Leung A, Sloan A, et al. Development and characterization of a Sin Nombre virus transmission model in *Peromyscus maniculatus.* Viruses. 2019;11:183. PubMed https://doi.org/10.3390/v11020183

2. Warner BM, Dowhanik S, Audet J, Grolla A, Dick D, Strong JE, et al. Hantavirus cardiopulmonary syndrome in Canada. Emerg Infect Dis. 2020;26:3020–4. PubMed https://doi.org/10.3201/eid2612.202808

3. Botten J, Mirowsky K, Kusewitt D, Ye C, Gottlieb K, Prescott J, et al. Persistent Sin Nombre virus infection in the deer mouse (*Peromyscus maniculatus*) model: sites of replication and strand-specific expression. J Virol. 2003;77:1540–50. PubMed https://doi.org/10.1128/JVI.77.2.1540-1550.2002

4. Warner BM, Jangra RK, Griffin BD, Stein DR, Kobasa D, Chandran K, et al. Oral vaccination with recombinant vesicular stomatitis virus expressing Sin Nombre virus glycoprotein prevents Sin Nombre virus transmission in deer mice. Front Cell Infect Microbiol. 2020;10:333. PubMed https://doi.org/10.3389/fcimb.2020.00333

Appendix Table. Detection of SNV antigenome in experimentally infected deer mice*

	Original infected sample		Experimental infection of deer mice			
			Positive strand–specific qRT-PCR positive/total samples, Ct value†			
Sample ID	Origin	qRT-PCR relative Ct value†	Lung	Liver	Kidney	Spleen
HAN266/23	Human	28.1	0/1(>35)	0/1(>35)	NA	NA
Vero-adapted SNV 77734	Vero cell line	22.0	2/3(32.1–34.7)	1/3(34.9)	3/3(31.4–33.6)	3/3(29.5–34.3)
		17.5	4/4(29.6–32.3)	1/4(33.6)	4/4(30.3–33.1)	3/4(29.7–31.8)
SNV 77734 DM	DM	20.7	1/1(20.1)	1/1(32.2)	1/1(25.7)	1/1(27.4)

*Ct, cycle threshold; DM, deer mouse; NA, not available; qRT-PCR, quantitative reverse transcription PCR; SNV, Sin Nombre virus.
†qRT-PCR threshold Ct <35.
